# Alterations in the respiratory tract microbiome in COVID-19: current observations and potential significance

**DOI:** 10.1186/s40168-022-01342-8

**Published:** 2022-10-05

**Authors:** Carter Merenstein, Frederic D. Bushman, Ronald G. Collman

**Affiliations:** 1 Department of Microbiology, Perelman School of Medicine, University of Pennsylvania, Philadelphia, PA 19104 USA; 2 Pulmonary, Allergy and Critical Care Division, Department of Medicine, University of Pennsylvania Perelman School of Medicine, Philadelphia, PA 19104 USA

## Abstract

SARS-CoV-2 infection causes COVID-19 disease, which can result in consequences ranging from undetectable to fatal, focusing attention on the modulators of outcomes. The respiratory tract microbiome is thought to modulate the outcomes of infections such as influenza as well as acute lung injury, raising the question to what degree does the airway microbiome influence COVID-19? Here, we review the results of 56 studies examining COVID-19 and the respiratory tract microbiome, summarize the main generalizations, and point to useful avenues for further research. Although the results vary among studies, a few consistent findings stand out. The diversity of bacterial communities in the oropharynx typically declined with increasing disease severity. The relative abundance of *Haemophilus* and *Neisseria* also declined with severity. Multiple microbiome measures tracked with measures of systemic immune responses and COVID outcomes. For many of the conclusions drawn in these studies, the direction of causality is unknown—did an alteration in the microbiome result in increased COVID severity, did COVID severity alter the microbiome, or was some third factor the primary driver, such as medication use. Follow-up mechanistic studies can help answer these questions.

Video Abstract

**Supplementary Information:**

The online version contains supplementary material available at 10.1186/s40168-022-01342-8.

## Introduction

COVID-19 was declared a pandemic by the World Health Organization on March 10, 2020, and has continued to cause disease and social disruption on an unprecedented scale despite the development of highly effective vaccines and both pharmacological and non-pharmaceutical interventions. COVID-19 is caused by infection with the SARS CoV-2 virus. COVID-19 can present with no symptoms, or with disease ranging from mild respiratory illness to severe lung injury, systemic inflammation, and ultimately death [1]. Given this wide range of outcomes, considerable effort has been devoted to predicting and explaining differential disease progression [2, 3]. One candidate modulator is the airway microbiome.

The microbiome of the human respiratory tract is diverse and heterogeneous and is associated with a wide range of diseases and phenotypes. The upper respiratory tract is inhabited by an abundant and complex microbiome, dominated by oral commensal taxa in healthy individuals. In contrast, the lower respiratory tract microbiome (below the vocal cords) in healthy individuals is generally quite low biomass, defined by competing dynamics of importation via microaspiration from the upper respiratory tract, and clearance via mucociliary activity and innate immune function, with likely some limited local microbial replication [4–6]. Thus, changes to the upper respiratory tract microbiome can affect not only local microbiome-host interactions, but those in the lower respiratory tract as well. Furthermore, in various disease states, the balance that maintains the low microbial biomass lung microbiome can be disrupted though increased entry to the lower respiratory tract, defective clearance, or increased microbial growth in the lower respiratory tract. The upper respiratory tract microbiome is altered in conditions such as advanced lung disease, HIV infection, vasculitis, and influenza, as well as by exposures such as smoking [7–12]. The lower respiratory tract (lung) microbiome is altered not only in suppurative lung diseases such as cystic fibrosis and pneumonia, but also in lung diseases not typically considered microbial, including asthma, COPD, and pulmonary fibrosis, as well as in exposures such as mechanical ventilation [7, 13–17].

Alterations in the upper respiratory microbiome have been associated with susceptibility to viral infection, specifically in influenza exposed individuals, and with disease severity in RSV infection [18, 19]. For instance, a household transmission study found increased abundance of *Streptococcus spp.* and *Prevotella salviae* was associated with reduced odds of influenza A infection [19]. Conversely, viral infection can alter the bacterial microbiome as well, resulting in potential bi-directional interactions [12]. Thus, there has been an intense interest in studies of the potential role of the airway microbiome in COVID-19.

One of the ways that the microbiota of the respiratory tract influences host health is via modulation of the immune system, both locally and systemically [20, 21]. This is of particular interest to COVID-19 because the immune response in COVID-19 has been shown to associate strongly with disease progression and outcome. In health, the lung immune tone is regulated by the physiological microbiome acquired through upper respiratory microaspiration [22, 23], and dysbiosis in the airway is associated with increased inflammation, for example, in asthma or exposure to cigarette smoke [13, 24]. Importantly, research over the past half-decade has suggested a role for the respiratory tract microbiome in modulating severity of outcomes in patients with or at risk of acute lung injury. Lung microbiome burden and composition correlate with local and systemic inflammation in patients with acute lung injury [25] and predict clinical outcome in mechanically ventilated critically ill patients with the acute respiratory distress syndrome (ARDS) [26]. Following trauma, lung microbiome composition is associated with the development of ARDS and has been postulated to mediate the effects of smoking on the risk of ARDS development [27]. Thus, the respiratory tract microbiome is linked with and may influence outcome via both local and systemic mechanisms in a variety of conditions that can result in severe lung injury.

In COVID-19, both immune suppression and runaway inflammation have been observed and can result in more severe disease, while protective immunity involves induction of both humoral and cell-mediated responses. Infection can induce several pro-inflammatory cytokines, such as IL-1B, IL-6, TNF, IL1RA, CXCL10/IP10, MIP-1α, and CCL2 [28, 29]. Several SARS CoV-2 proteins are capable of suppressing antiviral immunity by delaying the type I interferon response [29]. The microbiome has been identified as a modulator of immune responses and diseases in the airways [20, 22–24], leading to considerable interest in the interaction between COVID-19 and the airway microbiome.

The gut microbiome has also been studied as a potential modulator of COVID-19 disease severity, and several studies have described alterations in the gut microbiome in COVID-19 patients (reviewed in [30–32]). Consistent with a potential role, COVID-19 risk factors such as obesity and diabetes have profound impacts on the gut microbiome [33–35] and the gut microbiome is known to influence inflammatory conditions in the airway (referred to as the gut-lung axis) [36]. The relative contribution of gut versus respiratory microbiome communities on the respiratory tract is not clear, though a recent murine study suggested the inflammatory state of the lungs may be more closely linked to the local respiratory microbiome than the gut microbiome [37]. This review focuses on the respiratory microbiome and excludes studies of the gut microbiome in COVID-19 for the sake of clarity and focus; however, further study of the gut-lung axis is certainly warranted.

Here, we assess 56 studies examining the respiratory microbiome in COVID-19 patients, including studies of the oropharynx, nasopharynx, and lower airways/lungs (Supplemental Data 1). Studies were identified using Google Scholar searches for combinations of the following search terms: microbiome, COVID, COVID-19, SARS CoV-2, respiratory, airway, nasopharynx, nasopharyngeal, oropharynx, oropharyngeal, and lung. Only sequencing-based studies were included, whereas studies only performing bacterial culture were excluded. Studies vary considerably in subject population, disease severities, co-morbidities, medication use, and sample type. When possible, we have presented comparisons of similar findings between different studies in order to identify consistent changes and associations that may be more robust to study differences. These consistent associations offer high level commonality across a heterogeneous disease and can guide future studies in this nascent field.

## The oropharyngeal microbiome in COVID-19

**Fig. 1:**
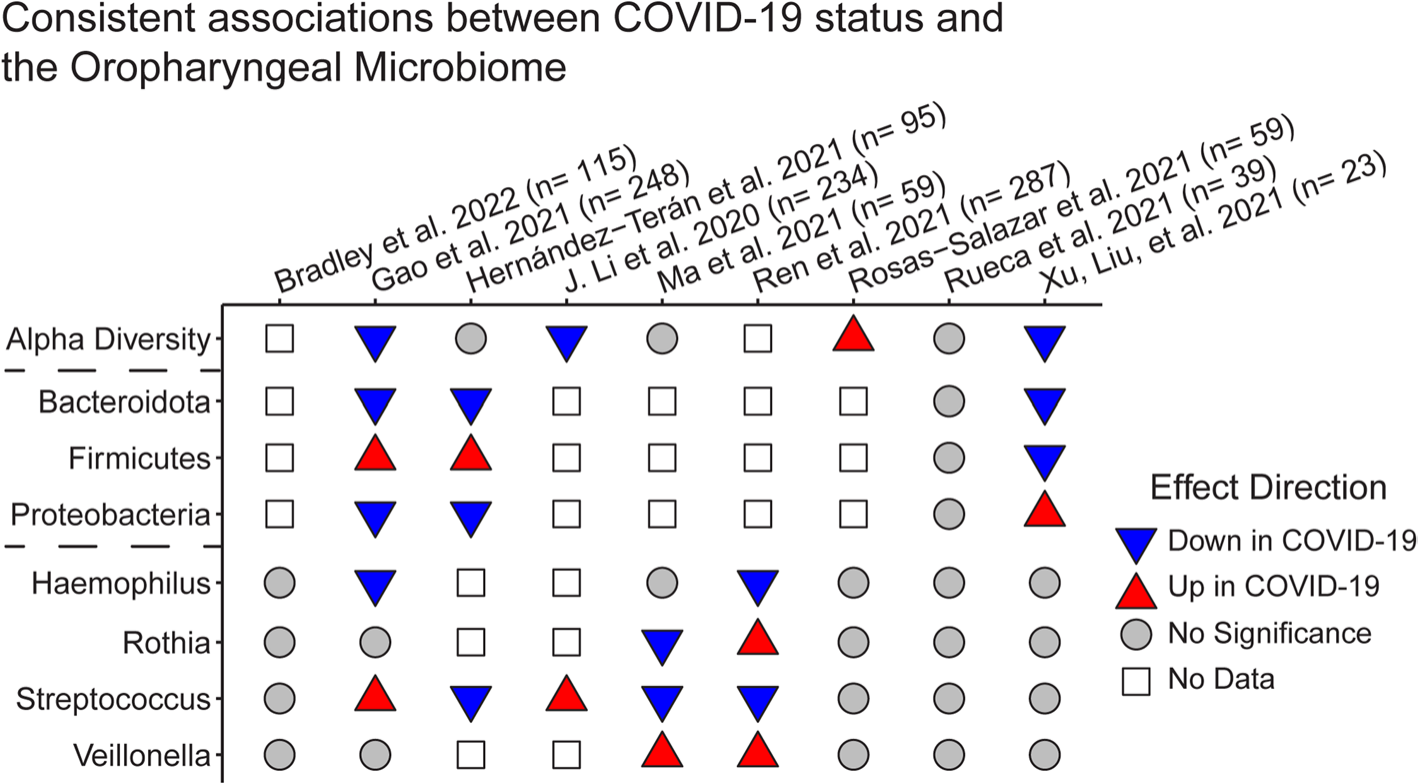
Associations of oropharyngeal microbiome features with COVID-19 relative to healthy controls. Analytical approaches and statistical methods varied among studies; results tabulated reflect the authors’ conclusions. Only studies that collected both COVID-19 and healthy controls are included; studies employing samples for either group from public data exclusively were not included. The sample size reports the number of COVID-19 patients and healthy controls. The results were filtered to emphasize findings consistent across more than one study

**Fig. 2:**
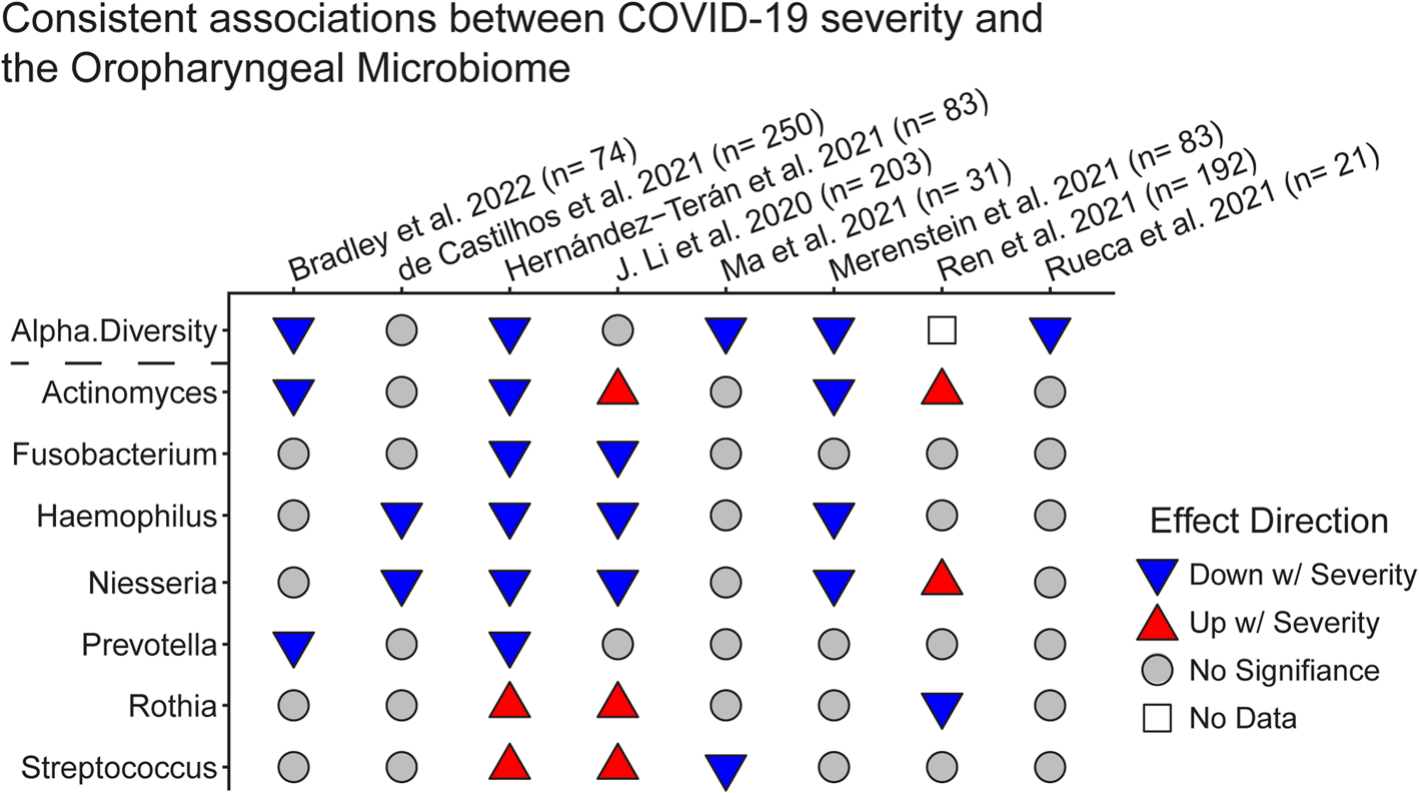
Associations between oropharyngeal microbiome features and COVID-19 severity. Analytical approaches and statistical methods varied among studies; results tabulated reflect the authors’ conclusions. Disease severity comparisons varied and included asymptomatic vs symptomatic, moderate vs severe, alive vs dead, and others. The sample size refers to the number of COVID-19 patients only

The upper respiratory tract microbiome is most often sampled using oropharyngeal (OP) or nasopharyngeal (NP) swabs. We identified 15 studies examining oropharyngeal microbial composition, including 9 studies directly comparing COVID-19 patients and healthy controls (Fig. 1) and 8 studies identifying microbiome associations with disease severity (Fig. 2). Across studies of COVID-19-infected individuals, the oropharyngeal microbiome is composed primarily of bacteria in the phyla *Proteobacteria, Firmicutes,* and *Bacteroidota* [38–40]. Specific taxa identified as abundant in the upper airway include *Prevotella, Streptococcus, Haemophilus, Neisseria, Viellonella,* and *Actinomyces*.

### The oropharyngeal microbiome is altered in COVID-19 relative to healthy controls

Most studies reported differences in the OP microbiome between COVID-19 patients and healthy controls, but there were few consistent changes identified across studies (Fig. 1). While each study identified taxa that were differentially abundant in COVID-19, there was no single taxa or microbiome metric that was consistently altered across the majority of the studies. However, the phylum *Bacteroidota* was less abundant in COVID-19 patients in three out of the four studies that presented phylum level compairisons [38, 41, 42]. *Proteobacteria* were less abundant and *Firmicutes* were increased in COVID-19 in two studies [38, 42], though this association was reversed in one study of children [41]. This association is somewhat unexpected, as relative abundance of *Proteobacteria* is increased in the airway in COPD, cystic fibrosis, and asthma [43]. At the genus level, *Haemophilus* was less abundant in COVID-19 patients in two studies, and *Veillonella* was more abundant across two studies.

Several taxa showed opposite associations in different studies; for example, *Streptococcus* abundance was negatively associated with COVID-19 in three studies [42, 44, 45] but positively associated in two studies [38, 46]. This could reflect differences in patient population or analytical techniques, species, or strain level differences or could suggest that abundances of these taxa are intrinsically noisy. Likewise, alpha diversity of the oropharyngeal microbiome was reduced in COVID-19 patients in three studies [38, 41, 46], increased in one [39], and unchanged in three [40, 42, 47]. Decreased diversity has been previously seen in influenza A infection [12], suggesting a possibly consistent response across viral respiratory infections.

The study by Gao and colleagues had a substantially larger sample size than others (140 healthy controls and 73 confirmed COVID-19) and identified the genera *Halomonas*, *Granulicatella, Leptotrichia*, and *Streptococcus* to be more abundant in COVID-19 patients, while *Neisseria*, *Prevotella*, *Alloprevotella, Fusobacterium*, and *Haemophilus* were less abundant [38]. It is possible therefore that with larger sample sizes some of these associations would be confirmed by other studies.

It is not possible to distinguish whether microbiome differences in COVID-19 patients compared with healthy people preceded SARS-CoV-2 infection or were a consequence of COVID-19 disease and treatments. For example, altered microbiome populations preceding exposure to SARS-CoV-2 could enhance susceptibility to infection or symptomatic disease. Alternatively, changes such as decreased diversity could reflect process that allowed one or a few bacterial species to grow out or that treatments such as antibiotics disrupted the community.

### Oropharyngeal microbiome associations with COVID-19 severity and outcome

Associations between the upper respiratory microbiome and COVID-19 severity are more consistent (Fig. 2). Alpha diversity commonly decreased as COVID-19 severity increased. At the genus level, *Haemophilus, Neisseria, Fusobacterium,* and *Prevotella* all showed reduced relative abundance in more severe patients across different studies. No taxa were consistently elevated in more severe COVID. This suggests a dysregulation of the respiratory microbiome wherein a diverse, healthy microbiome is associated with milder disease, and an overabundance of any number of different taxa is associated with more severe disease.

A major confounding factor is that therapeutic interventions may influence the microbiome in COVID-19 [48], with treatments such as antibiotics and mechanical intervention co-occurring with more severe disease (see Table 1). Bradley et al. sought to avoid this by sampling the oropharyngeal microbiome at hospital admission and predicting the future need for respiratory support [49]. This study found *P salviae, Eubacterium branchy*, *Actinomyces sp. S6 spd3*, and *Aggregatibacter sp. oral taxon 45* were all negatively associated with the need for respiratory support. A similarly predictive study by Ren et al. found *Streptococcus* relative abundance was increased at hospital admission in individuals who eventually recovered from COVID-19 compared to those who did not [45]. A major caveat of this second study is that samples were not taken specifically for microbiome analysis but were a byproduct of viral testing and so stored in viral transport media, which typically contains both growth nutrients and antibiotics, and has the potential to alter the microbial composition in samples. Still, these studies suggest that some changes in the upper airway microbiome are associated with COVID-19 severity in a treatment-independent manner.

**Table 1:** Challenges and considerations in COVID-19 respiratory microbiome studies

Challenge	Association with disease and microbiome	Mitigation
Antibiotic usage	More severe patients are more likely to receive antibiotics, making it difficult to differentiate between association w/disease severity and association w/antibiotic use.	Studies profiling patients prior to treatment avoid this effect (i.e., Bradley et al. [49]). Other studies with both COVID and non-COVID-19 patients receiving antibiotics can identify the effect of specific antibiotics, which can then be separated from the effects of COVID (i.e., Lloréns-Rico [48])
Mechanical ventilation	More severe patients require mechanical ventilation, which is known to impact the respiratory microbiome.	Similar mitigation techniques as used for high antibiotic use can apply to mechanical ventilation.
SARS CoV-2 variant	Some SARS CoV-2 variants may cause more or less severe disease, though the impact of variants on the respiratory microbiome is unknown.	The vast majority of studies here predate the emergence of any variants of concern, but in future studies, it will be important to sequence SARS CoV-2 genomes and report the variants included in microbiome studies to determine if there is an impact on the respiratory microbiome.
Sample storage in viral transport media	Swabs initially collected for SARS CoV-2 testing are often stored in VTM, which contains antibiotics and also nutrients that allow some bacteria to grow out.	Several studies sample the upper respiratory tract microbiome without storing in VTM (e.g., Braun et al., Mostafa et al. Hurst et al. [50–52]).
Prior immunity to SARS Cov-2	Patients with prior immunity via vaccination or prior infection are less likely to have severe COVID, but the impact of prior immunity on the microbiome are unknown.	No studies have yet reported the microbiome of COVID-19 patients with prior immunity. The impact can be mitigated by ensuring balanced enrollment of immunized and naïve patients at each level of disease severity in future microbiome studies.
Inconsistent analytical techniques	Each study uses different analysis methods to identify differential taxa in the microbiome. This is known to significantly impact which associations are identified.	Making data publicly available for reanalysis and meta-analysis would allow for standardization across studies. A minority of studies make sufficient data available (e.g., Gupta et al., Kullberg et al. [53, 54]).

Ultimately, studies of the oropharyngeal microbiome suggest reduced alpha diversity and reduced *Bacteroidota* in COVID-19 patients relative to healthy controls (Fig. 1). Within COVID-19, a consistent signature of lower alpha diversity, *Haemophilus, Neisseria, Actinomyces*, and *Prevotella* in more severe disease is seen across studies. Consistent findings across studies, despite differences populations, treatments, and comorbidities, suggest that these associations may be particularly strong. Future work is needed focusing on directionality and causality in these signature associations.

## The nasopharyngeal microbiome in COVID-19

**Fig. 3:**
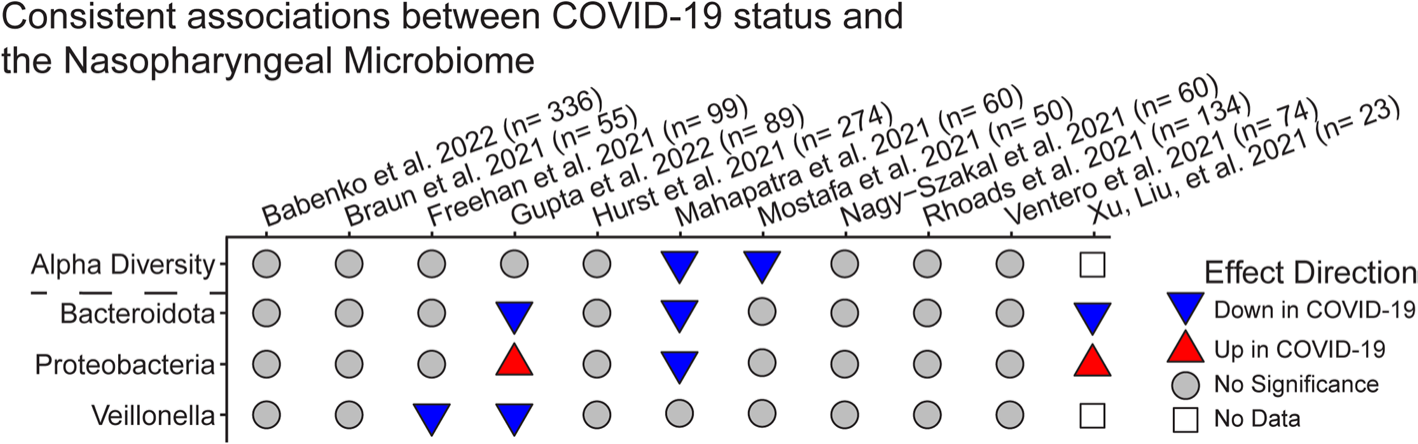
Associations of nasopharyngeal microbiome features with COVID-19, relative to healthy controls. Only studies that collected both COVID-19 and healthy controls are included; studies pulling samples for either group from public data exclusively were left out. The sample size reports the number of COVID-19 patients and healthy controls. The results were filtered to emphasize findings consistent across more than one study

**Fig. 4:**
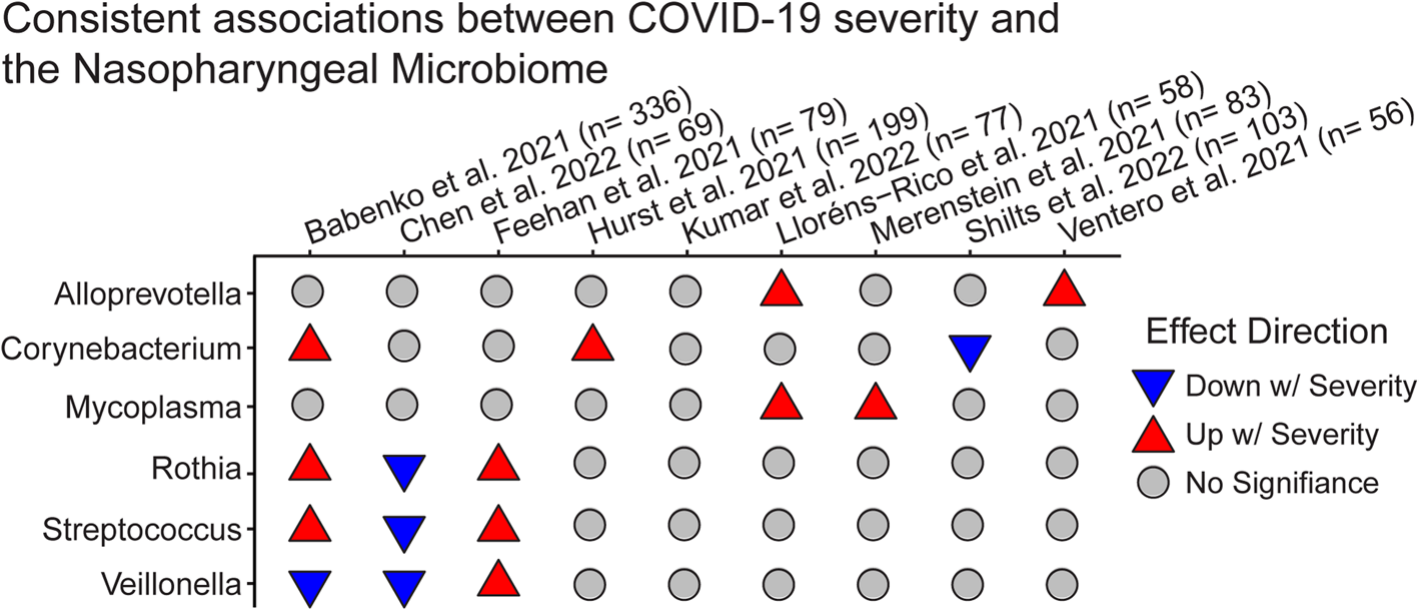
Associations between nasopharyngeal microbiome features and COVID-19 severity. Disease severity was defined differently across studies, ranging from asymptomatic vs symptomatic, to recovered vs deceased. Analytical approaches and statistical methods varied among studies; results tabulated reflect the authors’ conclusions. Disease severity comparisons varied, and included asymptomatic vs symptomatic, moderate vs severe, alive vs dead, and others. The sample size includes COVID-19 patients only

The nasopharyngeal microbiome is generally distinct from that of the rest of the respiratory tract, but is of interest in the context of COVID-19 as the nasal epithelium may be the first site of SARS CoV-2 infection. We identified 29 studies examining the nasopharyngeal microbiome in COVID-19 (Supplemental File 1), 12 of which examined differences between COVID-19 patients and healthy controls (Fig. 3), and 9 studied differences with COVID severity and outcomes (Fig. 4). Across studies, the nasopharyngeal microbiome was dominated by *Staphylococcus* and *Corynebacterium*, or by oral commensals similar to those found in the oropharynx, such as *Streptococcus, Prevotella,* and *Veillonella*. A major confounding factor for many nasopharyngeal studies was the storage of samples in viral transport media, as many studies looked at the microbiome using swabs that were initially collected for viral testing or sequencing [55–58].

### Nasopharyngeal microbiome shows few consistent differences between COVID-19 patients and healthy controls

Much like the oropharyngeal microbiome, there is little agreement between studies in differences between the nasopharyngeal microbiome in COVID-19 patients and healthy controls. Unlike the oropharynx, however, many studies find no significant differences at all with COVID-19 status [50, 55, 59, 60]. The most common association found across studies was a decrease in relative abundance of the phylum *Bacteroidota* [41, 56, 61], though this association was still not significant in most studies. Similarly, two studies found alpha diversity was lower in COVID-19 patients [51, 61], while eight studies found no significant difference [50, 52, 55, 56, 58–60, 62, 63], suggesting that the effect on alpha diversity is possibly negative and likely small.

Hurst and colleagues found the relative abundance of *Corynebacterium* was increased in children testing positive for COVID-19 relative to uninfected children from the same households who were also exposed to SARS CoV-2-infected individuals [52]. Conversely, Mostafa et al. find a decrease in relative abundance of *Corynebacterium* in COVID-19-positive adults compared to those that tested negative, suggesting the opposite association between this genus and COVID-19 susceptibility [51]. Since the studies were conducted in different age groups, age-related differences may possibly explain this disparity.

### Associations between nasopharyngeal microbiome and COVID-19 severity and outcomes

As with COVID-19 status, there are few consistent associations between the nasopharyngeal microbiome and COVID-19 severity, symptoms, or outcome (Fig. 4). The genus *Mycoplasma* was more abundant with increasing severity in two studies [48, 64] and was found in a third study to be strongly associated with presence of symptoms such as chest pain and fever [61]. Several taxa showed discordant associations with disease severity between studies, including *Fusobacterium, Rothia, Streptococcus, Veillonella, Prevotella*, and *Corynebacterium.* It is likely that differences in location, patient population, and analysis methods can account for discordant results, but further data is needed to identify consistent patterns for many of these taxa. For example, greater abundances of *Rothia* and *Streptococcus* were associated with increasing lung damage in a cohort in Russia [59], and with hospitalization in a study in the USA [58], but lower abundance was associated with severe disease among hospitalized patients from a Chinese cohort [65].

As discussed previously, COVID-19 severity is also unavoidably linked with various treatments that likely affect the nasopharyngeal microbiome composition, such as antibiotic use and mechanical ventilation. Unfortunately, in the nasopharyngeal microbiome, we identified no studies assessing severity of disease where sampling precedes treatment, making it impossible to separate this confounder. One study did attempt to quantify the impact of antibiotics, with Lloréns-Rico et al. finding the antibiotics meropenem/piperacillin-tazobactam and ceftriaxone significantly correlated with nasopharyngeal microbiome composition.

In sum, many current studies identify no significant associations between COVID-19 status or severity and the nasopharyngeal microbiome. The most consistent association across several studies is a reduction in *Bacteroidota* in COVID-19 patients relative to healthy controls. This suggests that any possible relationship between the nasopharyngeal microbiome and COVID-19 may be weak, heterogeneous, or easily obscured by common confounders.

## The lung microbiome in COVID-19

Adverse outcomes of SARS CoV-2 infection are associated with viral propagation from the upper respiratory tract to the lower airway and lungs. It is thus key to understand what effect the microbiome of these locations may have on the course of infection. There is no minimally invasive way to sample the lower airway or lung microbiome, so the lower airway has primarily been studied in critically ill COVID-19 patients who are intubated and on mechanical ventilation. This type of analysis has challenges, however, because the lung microbiome in intubated patients is profoundly altered compared to healthy people even in the absence of COVID-19 [17, 66, 67].

In a cohort of critically ill intubated COVID-19 patients, Merenstein and colleagues found lung microbiome specimens sampled by endotracheal aspirates showed low diversity compared to healthy lung samples and were frequently dominated by single taxa [64]. *Staphylococcus* and *Enterococcus* were particularly prominent in these patients. Tsitsiklis and coworkers also found reduced alpha diversity in intubated COVID-19 patients prior to the development of ventilator associated pneumonia relative to those that did not develop pneumonia [68]. Sulaiman et al. found a negative association between bacterial burden in the lungs and survival [69], while Kullberg et al. found similar association with successful extubation, even after accounting for clinical covariates such as antibiotic use [53]. This is consistent with previous findings in other forms of critical illness, where lung bacterial burden was negatively associated with ventilator-free days [26]. Kullberg and colleagues also found a significant association between beta diversity and bacterial burden, suggesting that outgrowth of bacteria in critically ill lungs may be driving compositional changes. Finally, Giabini et al. found low diversity in the lower airway of all mechanically ventilated COVID-19 patients, but it was not different from patients with non-COVID-19 pneumonia [70]. Again, this suggests that a low-diversity lower airway may not be a specific signature of COVID-19.

Giabini et al. further found lower relative abundances of oral commensals in COVID-19 patients, with a reduction in *Haemophilus influenzae*, *Veillonella dispar*, *Granulicatella* spp., *Porphyromonas* spp., and *Streptococcus* spp. and an increase in *Pseudomonas* spp*.* A study from the same hospital by Viciani et al. found increased colonization by *Candida* species in intubated COVID-19 patients relative to patients with non-COVID-19 pneumonia [71]. Sulaiman et al. found *Candida glabrata* enriched in the lungs of intubated patients with longer hospital stays [69], suggesting an altered fungal microbiome as well. Sulaiman et al. also found that reduced *P. oris* and increased *M. salivarium* in the lungs positively correlated with survival and length of stay in a mechanically ventilated cohort.

Zacharias and coworkers studied the microbiome of autopsied lung tissue and found reduced bacterial richness relative to deceased non-COVID-19 controls and reduced evenness in COVID-19 patients with pneumonia [72]. The authors identified dominant taxa in COVID-19 autopsy samples such as *Staphylococcus aureus, Enterococcus faecium*, or *Klebsiella pneumoniae*, as well as fungi like *Candida* spp. or the mold *Rhizopus microspores*, which they suggested could be secondary infections. These taxa align with those identified as dominant by Merenstein et al. and reflect well known opportunistic pathogens of the lungs. It should be noted, however, that these findings reflect only the sickest COVID-19 patients. Furthermore, these microbiome studies do not distinguish colonization of the lower respiratory tract by these organisms from clinically significant infection; while controversial, it has been suggested by a recent meta-analysis that bacterial co-infection in COVID-19 is relatively uncommon [73], although distinguishing colonization from bacterial superinfection is often challenging clinically. This is an important point, as a substantial proportion of mortality in other viral respiratory infections, particularly influenza, is caused by bacterial superinfection [74]. In seasonal influenza, a meta-analysis of confirmed cases by Klein et al. found bacterial co-infections in 23% of cases [75], and in the 2009 H1N1 pandemic, bacterial co-infections were identified in an estimated 29–55% of deaths [74].

Thus, the lungs of critically ill COVID-19 patients likely have reduced alpha diversity and increased bacterial burden relative to healthy or less sick COVID-19 patients, but not necessarily relative to other critically ill non-COVID-19 patients.

## Functional characterizations of the COVID-19 respiratory microbiome

Several studies have examined changes in functional capacity of the respiratory microbiome in COVID-19 based on microbiome gene content as reported in metagenomic sequence data or microbiome metatranscriptome data. Ma and colleagues found a higher potential for amino acid metabolism in the oropharyngeal metagenomes of COVID-19 patients compared to both healthy and flu-infected patients, specifically metabolism of valine, leucine, isoleucine, tyrosine, and phenylalanine [44]. Conversely, Li et al. suggested that amino acid metabolism was reduced in pharyngeal COVID-19 meta-transcriptomes, though this assertion was based on abundance of glutamate dehydrogenase which can have activities other than amino acid metabolism [46]. Membrane transport pathways also differed between these studies, being more abundant in healthy controls in Ma et al., but more abundant in COVID-19 patients in Li et al. These examples suggest that there may be considerable differences between functional potential (i.e., DNA) and functional activity (i.e., RNA).

Bradley et al. found LPS biosynthesis genes in the oropharynx were negatively predictive of the need for respiratory support, a somewhat surprising finding given the pro-inflammatory nature of LPS [49]. The authors suggest that this may be a result of a majority of the LPS genes coming from *Prevotella*, which has been hypothesized to have a less inflammatory LPS than more pathogenic bacteria. Indeed, the authors found a higher proportion of LPS coming from *Pseudomonas* in patients needing respiratory support. The implication that LPS source and structure is an important modulator of COVID-19 severity is especially interesting given reports that the SARS CoV-2 spike protein can directly bind to LPS [76] and that LPS in the blood is associated with COVID-19 severity [77].

Sulaiman and coworkers found no significant associations between metagenomic function and disease outcome, but did find metatranscriptomic differences [69]. Specifically, the authors found glycosylases, oxidoreductase activity, transporters, and the two-component system differentially upregulated in deceased patients. This suggests that the same bacteria may be changing function in more severe disease, though it is unclear which direction causality runs in these associations.

Multiple studies found increases in antimicrobial resistance genes in COVID-19 patients, involving a wide range of resistance mechanisms [44, 46, 62, 69]. Reflecting increased antibiotic usage in more severe patients, Li et al. found greater multidrug efflux pumps (SatA and SatB) in more severe patients [46]. Ma and coworkers found resistance to penam, penem, and cephalosporin antibiotics was most abundant and unique to COVID-19 compared to Flu B patients and healthy controls [44], while Nagy-Szakal et al. found macrolide resistance was most differentially abundant in COVID-19-positive samples [62].

## Associations of COVID-19 with microbial constituents other than bacteria

Respiratory tract fungal infections have been recognized as an important complication of COVID-19, which appear to be increased with the widespread use of dexamethasone and other corticosteroids and in patients treated with immunomodulators such as tocilizumab and baricitinib [78–80]. Most common is pulmonary aspergillosis, with notable reports of mucormycosis and multiple other fungi. Distinguishing infection from colonization is often difficult however, and evidence suggests that rates may vary across geographic regions [81].

Several metagenomic studies have found altered abundances in the fungal microbiome of COVID-19 patients, in both the upper and lower respiratory tract. Approximately 10% of hospitalized patients have positive respiratory fungal cultures across multiple studies, generally for *Candida* or *Aspergillus* [82, 83]. Two metagenomic studies found *Candida spp.* increased in the lower airway in more severe COVID-19 patients [69, 71]. Contrary to the bacterial microbiome, Hoque et al. found increased alpha diversity in the fungal microbiome of nasopharyngeal swabs in COVID-19 relative to healthy controls [84]. Nasopharyngeal fungal microbiome studies may be especially interesting since in addition to lung infection, Mucor spp. often initiate infection in the upper respiratory tract to invade the sinuses and brain, which has been reported in COVID-19 most commonly in India but globally as well [78, 85].

Viruses other than SARS CoV-2 were also detected in the airway of COVID-19 patients. Co-infections with respiratory viral pathogens appear to be rare, but *Influenza A, Influenza B, RSV, Human metapneumovirus, Parainfluenza, Rhinovirus,* and *Adenovirus* have all been reported in studies of COVID-19 patients [83]*.* In a small sample of critically ill patients, however, five of ten showed an increase in viral abundance, including viruses EBV, CMV, TTV, HSV, HPVB19, and JCV [86]. Additionally, an examination of the human endogenous retrovirus K found increased expression in endotracheal aspirate from intubated COVID-19 compared to intubated non-COVID-19 patients, but found no difference in outcome within COVID-19 [87]. Finally, Merenstein and colleagues found elevated levels of the commensal DNA virus *Anelloviridae* and recently described *Redondoviridae* in the oropharynx of intubated relative to non-intubated COVID-19 patients [64]. This suggests that critical illness and mechanical ventilation may impact abundance of already present human viruses, but it is yet unclear if this has any effect on the course of COVID-19.

Ultimately, more work is needed to examine changes in the fungal and viral microbiome in COVID-19. Many studies that collected metagenomic or metatranscriptomic data, which has the potential to identify fungal and viral genomes (in contrast to 16S rRNA gene sequencing, which is limited to bacteria), but only reported on the bacterial composition in their publications. Further analysis of these datasets would be fruitful for the study of the non-bacterial microbiome in COVID-19.

## The respiratory microbiome and associations with systemic immune responses

The immune response is critical for clearance of SARS CoV-2, and proper regulation is also needed to prevent a pathogenic hyperinflammatory response that is believed to contribute to severe outcomes. Several studies have identified associations between composition of the respiratory microbiome and systemic immune activation, although specific taxa and mechanisms that may mediate this are still unclear. Studies found associations between oropharyngeal microbiome composition and systemic lymphocyte counts: Merenstein et al. found alpha diversity to be positively associated with lymphocyte to neutrophil ratio [64]; Gao et al. found lymphocyte count negatively associated with *Leptotrichia* and *Streptococcus* but positively associated with 25 different 16S sequence groups (operational taxonomic units or OTUs) [38]; Hernández-Terán et al. found *Corynebacterium* associated with low lymphocyte counts [42]; and Ren et al. found *Streptococcus* positively correlated with lymphocyte counts [45]. Clinically, lymphocyte to neutrophil ratio is a key predictor of COVID-19 outcome, with decreased lymphocytes being associated with worse outcomes [88, 89].

Microbiome characteristics also correlated with inflammatory cytokine levels in the blood. Ren et al. found pro-inflammatory cytokines IL-1β, IL-6, IL-8, MCP-1, and IL-1ra were significantly correlated with overall oropharyngeal microbiome composition via principal component analysis, and several of these cytokines were more abundant in patients who died [45]. An assay of ex vivo cytokine production by Hursitoglu et al. also found more IL-1β production from the blood of COVID-19 patients with increased *Rothia* abundance in their nasopharynx, while anti-inflammatory cytokine IL-10 was associated with *Prophyromonas* abundance [90]. Surprisingly, no taxa from the genus *Haemophilus* were strongly associated with cytokine production or expression [45, 90], nor with neutrophil or lymphocyte abundance [38], despite being the taxa most consistently associated with COVID-19 severity across studies. Finally, one study found that elevated alveolar concentrations of inflammatory cytokines correlated with the concentration of bacterial and fungal DNA in bronchoalveolar lavages from COVID-19 ARDS patients [53], indicating a local mucosal immune response that is effected by the respiratory microbiome.

Together, these studies demonstrate links between the respiratory microbiome and immune and inflammatory features in COVID-19. One possibility for this observation is that the microbiome might be impacting disease outcomes through mechanisms mediated by systemic immune responses. Alternatively, if the microbiome changes are a consequence of COVID-19 infection and severity, it is possible that inflammatory features are driving microbiome changes. Thus, further study would be useful to understand the directionality and mechanisms of microbiome-immune associations in COVID-19.

## Comparisons of COVID-19 to influenza and other critical illnesses

To provide perspective, it is useful to consider associations between the respiratory microbiome and other viral illnesses. Prior to the COVID-19 pandemic, viral infections were known to have bidirectional relationships with the respiratory microbiome. For instance, abundance of *Streptococcus spp.* and *Prevotella salviae* associated with reduced odds of influenza A infection in exposed individuals [19], though several other species of *Prevotella* were positively associated with influenza A susceptibility in a similar household transmission study [18]. These relationships appeared to be virus specific, however, with different taxa associated with Influenza A and B susceptibility [19].

Viral infection can alter the bacterial microbiome as well; for example, Kaul and colleagues found increased *Pseudomonas* in the nasopharynx of influenza A patients compared to non-infected controls [12]. This too appears virus-specific, as a challenge trial in adults inoculated with respiratory syncytial virus found no alterations in the respiratory microbiome after infection [91].

A few studies have directly compared the microbiome of COVID-19 patients with influenza patients. Ma and coworkers studied the oropharyngeal microbiome and found both a common viral signature relative to healthy controls and several associations that were specific to COVID-19 patients [44]. An increase in *Viellonella* was seen in both influenza and COVID-19 patients relative to controls, but was significantly higher in COVID-19 relative to influenza patients, suggesting that even shared associations may differ in their magnitude or significance in a given viral infection. A similar study by Rattanaburi et al. compared the nasopharyngeal microbiome of influenza A, influenza B, and COVID-19 patients [92]. Both groups of influenza patients had microbiomes dominated by Proteobacteria, while the COVID-19 group was more similar to the non-COVID-19 non-influenza group dominated by Firmicutes and Bacteroidota. A caveat of this study, however, was that influenza samples were collected several years prior to COVID-19 samples, introducing potential batch effects.

In addition to other viral illnesses, alterations in the microbiome of critically ill patients from other causes may resemble COVID-19 alterations. Fortunately, several studies have compared COVID-19 patients to those with other forms of severe pneumonia or other diseases requiring intubation. One such study compared critically ill non-COVID-19 patients to COVID-19 patients of varying degrees of illness and found significantly lower Proteobacteria in non-COVID-19 patients compared to all groups of COVID-19 patients, in the oropharynx [64]. Miao et al. found lower airway microbiomes of intubated COVID-19 patients to be significantly different from intubated non-COVID-19 patients via PERMANOVA test, but both groups were much more closely clustered to each other than to non-intubated patients, either healthy or with other viral pneumonia [86].

Sputum meta-transcriptomes from COVID-19 patients also showed lower alpha diversity compared to a large cohort of non-COVID-19 pneumonia patients, suggesting that the consistent signature of low airway diversity seen in many COVID-19 studies is not necessarily common to all severe respiratory infections [93]. Additionally, Viciani and colleagues found increased *Candida* colonization in COVID-19 lower airways relative to non-COVID-19 pneumonia patients, again suggesting that previously discussed associations are not general to all pneumonias [71].

Direct comparisons between COVID-19 and other airway diseases are complicated by differences in treatments and patient demographics. It is thus possible that some signatures that appear unique to COVID-19 are rather caused by some combination of general response to critical lung injury and specific response to antibiotics, steroids, or intubation that may be more common in COVID-19. Nevertheless, most direct comparisons show substantial differences between COVID-19 patients and those infected with other viruses or experiencing other forms of critical illness, and it is likely that much of the respiratory microbiome response to COVID-19 is unique to that disease.

## Limitations and confounding factors of respiratory microbiome studies in COVID19

As discussed throughout the previous sections, there are important limitations in respiratory microbiome studies of COVID-19 to date (Table 1). First, many studies rely on samples initially collected for viral detection that were stored in viral transport media, which typically contains both nutrients that might spur bacterial growth and antibiotics that might skew its composition. It is likely that in some of these studies, the composition of the microbiome continued to change after collection and may not accurately reflect in vivo communities. Fortunately, most of these studies used the same procedures for all samples, possibly retaining some ability for within-study comparisons, even if in absolute terms the data may be less accurate.

The second major source of bias in these studies is the effect that COVID-19 treatments may have on microbiome composition [48]. As discussed earlier, antibiotic use and mechanical ventilation can cause major shifts in the respiratory microbiome. Antibiotics and invasive mechanical ventilation are commonly employed in severe COVID-19 patients, with a large proportion of patients receiving antibiotics [40, 64]. In the study by de Castilhos and colleagues, antibiotic use significantly influenced the oropharyngeal microbiome and led to increased dysbiosis, but alpha diversity was not affected [40]. Lloréns-Rico and colleagues found that the nasopharyngeal microbiome composition was significantly correlated with length of ICU stay and use of several specific antibiotics (meropenem/piperacillin-tazobactam, and ceftriaxone), though neither number of antibiotics nor ongoing antibiotic use as a binary variable were associated with composition [48].

Mechanical ventilation also alters the respiratory microbiome, allowing for the outgrowth of opportunistic pathogens and, in some cases, ventilator-associated pneumonia [17, 66]. Given the association between COVID-19 severity and the need for ventilation, further work may be needed to separate the effects of the disease from its treatments. This is a substantial limitation in most studies of the respiratory microbiome and disease severity, as these disruptive treatments are inherently correlated with disease severity. Studies with non-COVID-19 patients also in similar critical care settings can begin to separate these effects from the impact of COVID-19, and studies collecting samples prior to treatment will avoid these confounders.

The timing of sample collection also limits interpretation of many studies, because it is unclear whether the microbiome is affecting COVID-19 susceptibility and severity, or whether alterations are a consequence of infection. We identified no studies with respiratory microbiome samples preceding SARS-CoV-2 infection, making it difficult to conclusively establish timing, let alone causality, for any of the associations presented in these papers.

Finally, work in this field would be greatly aided by better data management and public archiving of data to allow use of existing data to its full potential. Here, we identified over 50 studies examining the respiratory microbiome in COVID-19, with thousands of samples total. Unfortunately, the majority of studies have not yet made their data publicly available, and most of those that are available lack sufficient metadata for to allow meta-analysis (i.e., patients cannot be matched to samples in public databases). This makes reanalysis of multiple data sets using a single consistent approach impossible. If future studies make both raw data and metadata available, it may be possible to pool studies and collect sufficient sample sizes to answer questions beyond what individual efforts are able to address.

## Conclusions and potential significance of COVID-19/microbiome associations

Despite the limitations associated with studying a rapidly evolving disease in the midst of a global pandemic, current evidence suggests that the respiratory microbiome is altered in COVID-19 compared to healthy people and that microbiome changes are associated with disease severity, though the literature is in only partial agreement about specific changes involved. Multiple factors differ between studies, including timing of samples, method of sampling, disease severity, treatments, and patient populations, probably accounting at least in part for the variability. So far results seem to be cohort specific. Nevertheless, there is some convergence on particular associations, especially in the oropharyngeal microbiome. Alpha diversity is consistently lower in more severe COVID patients, as are relative abundances of the genera *Haemophilus* and *Neisseria*. These findings suggest a pattern of dysbiosis wherein a healthy, diverse community of oral commensals is replaced by an outgrowth of a small number of taxa, which vary from patient to patient. In the nasopharyngeal microbiome, few consistent associations were identified, and several studies found no significant differences with COVID-19 status or severity.

Particularly compelling associations between the respiratory microbiome and COVID-19 involve systemic immune response. Multiple studies found microbiome features associated with systemic immunity, including factors such as lymphocyte to neutrophil ratio, that have strong associations with COVID-19 outcomes. Specific taxa involved in these interactions vary from study to study, again suggesting the need for more diverse patient populations and larger cohorts to identify robust associations. Future work is critical to determine if the microbiome is changing because of changes in the immune environment, or if bacteria in the airway might push the immune system toward a particular response. Additionally, we have no studies thus far of the microbiome in immunized populations, or those with prior infection, and further work is needed to understand how the respiratory microbiome may influence long-term adaptive immunity to SARS CoV-2.

Key next steps for COVID-19 respiratory microbiome research are to determine causality and mechanisms for associations identified so far. Are respiratory microbiome changes in COVID-19 entirely a consequence of infection and/or its treatments? Or does respiratory tract dysbiosis precede SARS-CoV-2 infection and perhaps contribute to infection susceptibility and severity of disease? Regarding whether microbiome alterations might precede and contribute to COVID-19 severity, there is limited information known about the respiratory microbiome in conditions associated with high COVID-19 risk. Alterations of the upper respiratory tract microbiome have been found in healthy elderly people [94], but whether individuals with diabetes, obesity, or other risk factors for severe COVID-19 have patterns of dysbiosis is unknown. Even if COVID-19 disease is primarily driving changes in the microbiome, it will be important to understand whether early changes impact later stages of the disease, including inflammatory response, acute lung injury, and long-COVID.

How might the respiratory tract microbiome mechanistically impact COVID-19 infection? The upper respiratory tract is the site of initial SARS-CoV-2 infection, so microbiome effects on the local immune milieu might regulate virus replication. Additionally, the SARS-CoV-2 cellular receptor ACE2 is an interferon-stimulated gene [95], so its expression could also be linked to microbiome influences. Additionally, since the upper respiratory tract microbiome seeds the lower respiratory tract [4–6], similar effects could also modulate infection in the lung. Finally, lower respiratory microbiome links to the development of acute lung injury following other types of insults are thought to be mediated by local and systemic inflammatory pathways [26, 27], which may be operative in SARS-CoV-2 infection also.

Thus, respiratory microbiome studies of people at high risk for severe COVID-19 are needed to address direction and causality. Earlier and more systematic microbiome sampling in COVID-19 will be important, and with continued circulation of COVID-19, prospective studies may be possible to determine how the respiratory microbiome in uninfected individuals affects the eventual course of subsequent infection. Longitudinal studies in diverse cohorts that span from before infection to after recovery would aid significantly in determining causality in these associations. Possible effects of viral variants need to be addressed more fully. Studies relating specific microbes with respiratory mucosal immune features and gene expression are also needed. Additionally, in vitro studies are essential to determine mechanisms that link the respiratory microbiome to features seen in COVID-19 infection, as well as mechanistic studies in animal models. Key steps include identifying how different COVID-19 associated bacteria may change gene expression in airway epithelial cells or cytokine production in mucosal immune cells.

Together, such studies could suggest whether manipulating the respiratory tract microbiome might be a new therapeutic target for prevention or treatment of COVID-19 or whether microbiome features might be useful as a biomarker of COVID-19 risk.

## Supplementary Information

**Additional file 1.** Datasheet.

## Data Availability

A complete list of publications reviewed in this paper is available in supplemental data file 1.

## References

[1] Li L (2020). COVID-19 patients’ clinical characteristics, discharge rate, and fatality rate of meta-analysis. J Med Virol.

[2] Izcovich A (2020). Prognostic factors for severity and mortality in patients infected with COVID-19: a systematic review. PLoS One.

[3] Gallo Marin B (2021). Predictors of COVID-19 severity: a literature review. Rev Med Virol.

[4] Cui L (2014). The microbiome and the lung. Ann Am Thorac Soc.

[5] Whiteside SA, McGinniss JE, Collman RG (2021). The lung microbiome: progress and promise. J Clin Invest.

[6] Charlson ES (2011). Topographical continuity of bacterial populations in the healthy human respiratory tract. Am J Respir Crit Care Med.

[7] Morris A (2013). Comparison of the respiratory microbiome in healthy nonsmokers and smokers. Am J Respir Crit Care Med.

[8] Charlson ES (2010). Disordered microbial communities in the upper respiratory tract of cigarette smokers. PLoS One.

[9] Simon-Soro A (2019). Upper respiratory dysbiosis with a facultative-dominated ecotype in advanced lung disease and dynamic change after lung transplant. Ann Am Thorac Soc.

[10] Beck JM (2015). Multicenter comparison of lung and oral microbiomes of HIV-infected and HIV-uninfected individuals. Am J Respir Crit Care Med.

[11] Rhee RL (2018). Characterisation of the nasal microbiota in granulomatosis with polyangiitis. Ann Rheum Dis.

[12] Kaul D (2020). Microbiome disturbance and resilience dynamics of the upper respiratory tract during influenza A virus infection. Nat Commun.

[13] Huang YJ (2015). The respiratory microbiome and innate immunity in asthma. Curr Opin Pulm Med.

[14] Surette MG (2014). The cystic fibrosis lung microbiome. Ann Am Thorac Soc.

[15] Pragman AA, Kim HB, Reilly CS, Wendt C, Isaacson RE (2012). The lung microbiome in moderate and severe chronic obstructive pulmonary disease. PLoS One.

[16] Twigg HL (2016). Effect of advanced HIV infection on the respiratory microbiome. Am J Respir Crit Care Med.

[17] Fromentin M, Ricard J-D, Roux D (2021). Respiratory microbiome in mechanically ventilated patients: a narrative review. Intensive Care Med..

[18] Lee KH (2019). The respiratory microbiome and susceptibility to influenza virus infection. PLoS One.

[19] Tsang TK (2020). Association between the respiratory microbiome and susceptibility to influenza virus infection. Clin Infect Dis.

[20] Huffnagle GB, Dickson RP, Lukacs NW (2017). The respiratory tract microbiome and lung inflammation: a two-way street. Mucosal Immunol.

[21] Invernizzi R, Lloyd CM, Molyneaux PL (2020). Respiratory microbiome and epithelial interactions shape immunity in the lungs. Immunology.

[22] Segal LN (2013). Enrichment of lung microbiome with supraglottic taxa is associated with increased pulmonary inflammation. Microbiome.

[23] Segal LN (2016). Enrichment of the lung microbiome with oral taxa is associated with lung inflammation of a Th17 phenotype. Nat Microbiol.

[24] Li K (2019). Dysbiosis of lower respiratory tract microbiome are associated with inflammation and microbial function variety. Respir Res.

[25] Dickson RP (2016). Enrichment of the lung microbiome with gut bacteria in sepsis and the acute respiratory distress syndrome. Nat Microbiol.

[26] Dickson RP (2020). Lung microbiota predict clinical outcomes in critically ill patients. Am J Respir Crit Care Med.

[27] Panzer AR (2018). Lung microbiota is related to smoking status and to development of acute respiratory distress syndrome in critically ill trauma patients. Am J Respir Crit Care Med.

[28] García LF (2020). Immune response, inflammation, and the clinical spectrum of COVID-19. Front Immunol.

[29] Ramasamy S, Subbian S (2021). Critical determinants of cytokine storm and type I interferon response in COVID-19 pathogenesis. Clin Microbiol Rev.

[30] Yamamoto S (2021). The human microbiome and COVID-19: a systematic review. PLoS One.

[31] Segal JP (2020). The gut microbiome: an under-recognised contributor to the COVID-19 pandemic?. Ther Adv Gastroenterol.

[32] Najmi N, Megantara I, Andriani L, Goenawan H, Lesmana R (2022). Importance of gut microbiome regulation for the prevention and recovery process after SARS-CoV-2 respiratory viral infection (Review). Biomed Rep.

[33] Vallianou NG, Stratigou T, Tsagarakis S (2018). Microbiome and diabetes: where are we now?. Diabetes Res Clin Pract.

[34] Singer-Englar T, Barlow G, Mathur R (2019). Obesity, diabetes, and the gut microbiome: an updated review. Expert Rev Gastroenterol Hepatol.

[35] Castaner O (2018). The gut microbiome profile in obesity: a systematic review. Int J Endocrinol.

[36] Dang AT, Marsland BJ (2019). Microbes, metabolites, and the gut–lung axis. Mucosal Immunol.

[37] Dickson RP (2018). The lung microbiota of healthy mice are highly variable, cluster by environment, and reflect variation in baseline lung innate immunity. Am J Respir Crit Care Med.

[38] Gao M (2021). Characterization of the human oropharyngeal microbiomes in SARS-CoV-2 infection and recovery patients. Adv Sci.

[39] Rosas-Salazar C (2021). SARS-CoV-2 infection and viral load are associated with the upper respiratory tract microbiome. J Allergy Clin Immunol.

[40] de Castilhos J, et al. Severe dysbiosis and specific Haemophilus and Neisseria signatures as hallmarks of the oropharyngeal microbiome in critically ill COVID-19 patients. Clin Infect Dis. 2021;25:ciab902. https://pubmed.ncbi.nlm.nih.gov/34694375/. 10.1093/cid/ciab902 PMC8586732 34694375

[41] Xu R (2021). Progressive deterioration of the upper respiratory tract and the gut microbiomes in children during the early infection stages of COVID-19. J Genet Genomics.

[42] Hernández-Terán A (2021). Dysbiosis and structural disruption of the respiratory microbiota in COVID-19 patients with severe and fatal outcomes. Sci Rep.

[43] Caverly LJ, Huang YJ, Sze MA (2019). Past, present, and future research on the lung microbiome in inflammatory airwaydisease. Chest.

[44] Ma S (2021). Metagenomic analysis reveals oropharyngeal microbiota alterations in patients with COVID-19. Signal Transduct Target Ther.

[45] Ren L (2021). Dynamics of the upper respiratory tract microbiota and its association with mortality in COVID-19. Am J Respir Crit Care Med.

[46] Li J, et al. Assessment of microbiota in the gut and upper respiratory tract associated with SARS-CoV-2 infection. SSRN (preprint). 2020. Preprint at 10.2139/ssrn.3702488. 10.1186/s40168-022-01447-0 PMC9982190 36869345

[47] Rueca M (2021). Investigation of nasal/oropharyngeal microbial community of COVID-19 patients by 16S rDNA sequencing. Int J Environ Res Public Health.

[48] Lloréns-Rico V, et al. Clinical practices underlie COVID-19 patient respiratory microbiome composition and its interactions with the host. medRxiv (preprint). 2021: 2020.12.23.20248425. Preprint at 10.1101/2020.12.23.20248425. 10.1038/s41467-021-26500-8 PMC8556379 34716338

[49] Bradley ES, et al. Oropharyngeal microbiome profiled at admission is predictive of the need for respiratory support among COVID-19 patients. medRxiv (preprint). 2022: 2022.02.28.22271627 Preprint at 10.1101/2022.02.28.22271627. 10.3389/fmicb.2022.1009440 PMC9561819 36246273

[50] Braun T (2021). SARS-CoV-2 does not have a strong effect on the nasopharyngeal microbial composition. Sci Rep.

[51] Mostafa HH (2020). Metagenomic next-generation sequencing of nasopharyngeal specimens collected from confirmed and suspect COVID-19 patients. mBio.

[52] Hurst JH, et al. Age-related changes in the upper respiratory microbiome are associated with SARS-CoV-2 susceptibility and illness severity. medRxiv (preprint). 2021:2021.03.20.21252680 Preprint at 10.1101/2021.03.20.21252680.

[53] Kullberg RFJ, et al. Lung microbiota of critically ill COVID-19 patients are associated with non-resolving acute respiratory distress syndrome. Am J Respir Crit Care Med. 2022. 10.1164/rccm.202202-0274OC. 10.1164/rccm.202202-0274OC PMC9799265 35616585

[54] Gupta A (2022). Nasopharyngeal microbiome reveals the prevalence of opportunistic pathogens in SARS-CoV-2 infected individuals and their association with host types. Microbes Infect.

[55] Shilts MH (2022). Severe COVID-19 is associated with an altered upper respiratory tract microbiome. Front Cell Infect Microbiol.

[56] Gupta VK (2020). A predictive index for health status using species-level gut microbiome profiling. Nat Commun.

[57] Kolhe R (2021). Alteration in nasopharyngeal microbiota profile in aged patients with COVID-19. Diagnostics.

[58] Feehan AK (2021). Nasopharyngeal microbiome community composition and structure is associated with severity of COVID-19 disease and breathing treatment. Appl Microbiol.

[59] Babenko V, et al. Analysis of the upper respiratory tract microbiota in mild and severe COVID-19 patients. bioRxiv (preprint). 2021: 2021.09.20.461025 Preprint at 10.1101/2021.09.20.461025.

[60] Ventero MP (2021). Nasopharyngeal microbial communities of patients infected with SARS-CoV-2 that developed COVID-19. Front Microbiol.

[61] Mahapatra S, et al. Nanopore 16S rRNA sequencing reveals alterations in nasopharyngeal microbiome and enrichment of Mycobacterium and Mycoplasma in patients with COVID 19. medRxiv (preprint). 2021: 2021.11.10.21266147. Preprint at 10.1101/2021.11.10.21266147.

[62] Nagy-Szakal D, et al. Targeted hybridization capture of SARS-CoV-2 and metagenomics enables genetic variant discovery and nasal microbiome insights. Microbiol Spectr. 9:e00197–21. 10.1128/Spectrum.00197-21 PMC8557865 34468193

[63] Rhoades NS (2021). Acute SARS-CoV-2 infection is associated with an increased abundance of bacterial pathogens, including Pseudomonas aeruginosa in the nose. Cell Rep.

[64] Merenstein C (2021). Signatures of COVID-19 severity and immune response in the respiratory tract microbiome. mBio.

[65] Chen J, et al. Comparison of the respiratory tractmicrobiome in hospitalized COVID-19 patients with different disease severity. ResearchSquare (preprint). 2022. Preprint at 10.21203/rs.3.rs-1237564/v1.

[66] Kelly BJ (2016). Composition and dynamics of the respiratory tract microbiome in intubated patients. Microbiome.

[67] Zakharkina T (2017). The dynamics of the pulmonary microbiome during mechanical ventilation in the intensive care unit and the association with occurrence of pneumonia. Thorax.

[68] Tsitsiklis A, et al. Impaired immune signaling and changes in the lung microbiome precede secondary bacterial pneumonia in COVID-19. medRxiv (preprint). 2021: 2021.03.23.21253487 Preprint at 10.1101/2021.03.23.21253487.

[69] Sulaiman I (2021). Microbial signatures in the lower airways of mechanically ventilated COVID-19 patients associated with poor clinical outcome. Nat Microbiol.

[70] Gaibani P (2021). The lower respiratory tract microbiome of critically ill patients with COVID-19. Sci Rep.

[71] Viciani E (2022). Critically ill patients with COVID-19 show lung fungal dysbiosis with reduced microbial diversity in patients colonized with Candida spp. Int J Infect Dis.

[72] Zacharias M, et al. Host and microbiome features of secondary infections in lethal covid-19. medRxiv (preprint). 2022: 2022.02.18.22270995. Preprint at 10.1101/2022.02.18.22270995. 10.1016/j.isci.2022.104926 PMC9374491 35992303

[73] Langford BJ (2020). Bacterial co-infection and secondary infection in patients with COVID-19: a living rapid review and meta-analysis. Clin Microbiol Infect.

[74] Morris DE, Cleary DW, Clarke SC (2017). Secondary bacterial infections associated with influenza pandemics. Front Microbiol.

[75] Klein EY (2016). The frequency of influenza and bacterial coinfection: a systematic review and meta-analysis. Influenza Other Respir Viruses.

[76] Petruk G (2020). SARS-CoV-2 spike protein binds to bacterial lipopolysaccharide and boosts proinflammatory activity. J Mol Cell Biol.

[77] Kruglikov IL, Scherer PE (2021). Preexisting and inducible endotoxemia as crucial contributors to the severity of COVID-19 outcomes. PLoS Pathog.

[78] Hoenigl M, et al. The emergence of COVID-19 associated mucormycosis: a review of cases from 18 countries. Lancet Microbe. 2022. 10.1016/S2666-5247(21)00237-8. 10.1016/S2666-5247(21)00237-8 PMC8789240 35098179

[79] Gangneux J-P (2022). Fungal infections in mechanically ventilated patients with COVID-19 during the first wave: the French multicentre MYCOVID study. Lancet Respir Med.

[80] Prattes J (2022). Risk factors and outcome of pulmonary aspergillosis in critically ill coronavirus disease 2019 patients-a multinational observational study by the European Confederation of Medical Mycology. Clin Microbiol Infect Off Publ Eur Soc Clin Microbiol Infect Dis.

[81] Verweij PE (2021). Taskforce report on the diagnosis and clinical management of COVID-19 associated pulmonary aspergillosis. Intensive Care Med.

[82] Song G, Liang G, Liu W (2020). Fungal co-infections associated with global COVID-19 pandemic: a clinical and diagnostic perspective from China. Mycopathologia.

[83] Musuuza JS (2021). Prevalence and outcomes of co-infection and superinfection with SARS-CoV-2 and other pathogens: a systematic review and meta-analysis. PLoS One.

[84] Hoque MN, et al. Metagenomic analysis reveals the abundance and diversity of opportunistic fungal pathogens in the nasopharyngeal tract of COVID-19 patients. 2022: 2022.02.17.480819 Preprint at 10.1101/2022.02.17.480819.

[85] Danion F (2022). Coronavirus disease 2019-associated mucormycosis in France: a rare but deadly complication. Open Forum Infect Dis.

[86] Miao Q (2021). Evaluation of superinfection, antimicrobial usage, and airway microbiome with metagenomic sequencing in COVID-19 patients: a cohort study in Shanghai. J Microbiol Immunol Infect.

[87] Temerozo JR (2022). Human endogenous retrovirus K in the respiratory tract is associated with COVID-19 physiopathology. Microbiome.

[88] Chan AS, Rout A (2020). Use of neutrophil-to-lymphocyte and platelet-to-lymphocyte ratios in COVID-19. J Clin Med Res.

[89] Jimeno S (2021). Prognostic implications of neutrophil-lymphocyte ratio in COVID-19. Eur J Clin Invest.

[90] Hursitoglu M (2022). In-vitro cytokine production and nasopharyngeal microbiota composition in the early stage of COVID-19 infection. Cytokine.

[91] Cuthbertson L, et al. Resilience of the respiratory microbiome in controlled adult RSV challenge study. Eur Respir J. 2022;59(1):2101932. 10.1183/13993003.01932-2021. 10.1183/13993003.01932-2021 PMC8754103 34711536

[92] Rattanaburi S (2022). Bacterial microbiota in upper respiratory tract of COVID-19 and influenza patients. Exp Biol Med.

[93] Zhang H, et al. Metatranscriptomic characterization of coronavirus disease 2019 identified a host transcriptional classifier associated with immune signaling. Clin Infect Dis. 2020. 10.1093/cid/ciaa663. 10.1093/cid/ciaa663 PMC7314197 32463434

[94] Whelan FJ (2014). The loss of topography in the microbial communities of the upper respiratory tract in the elderly. Ann Am Thorac Soc.

[95] Ziegler CGK (2020). SARS-CoV-2 Receptor ACE2 is an interferon-stimulated gene in human airway epithelial cells and is detected in specific cell subsets across tissues. Cell.

